# When the mother-in-law is just as good—Differential mortality of reproductive females by family network composition

**DOI:** 10.1371/journal.pone.0193252

**Published:** 2018-03-01

**Authors:** Kai Pierre Willführ, Johannes Johow, Eckart Voland

**Affiliations:** 1 Max-Planck-Institute for Demographic Research, Rostock, Germany; 2 Carl von Ossietzky University, Oldenburg, Germany; 3 Independent Researcher, Gießen, Germany; 4 Institute for Philosophy, University of Gießen, Germany; London School of Hygiene and Tropical Medicine, UNITED KINGDOM

## Abstract

Motivated by the cooperative breeding hypothesis, we investigate the effect of having kin on the mortality of reproductive women based on family reconstitutions for the Krummhörn region (East Frisia, Germany, 1720–1874). We rely on a combination of Cox clustered hazard models and hazard models stratified at the family level. In order to study behavior-related effects, we run a series of models in which only kin who lived in the same parish are considered. To investigate structural, non-behavior-related effects, we run a different model series that include all living kin, regardless their spatial proximity. We find that women of reproductive age who had a living mother had a reduced mortality risk. It appears that having living sisters had an ambivalent impact on women’s mortality: i.e., depending on the socioeconomic status of the family, the effect of having living sisters ranged between representing a source of competition and representing a source of support. Models which are clustered at the family level suggest that the presence of a living mother-in-law was associated with reduced mortality among her daughters-in-law especially among larger-scale farm families. We interpret this finding as a consequence of augmented consanguineous marriages among individuals of higher social strata. For instance, in first cousin marriages, the mother-in-law could also be a biological aunt. Thus, it appears that among the wealthy elite, the genetic in-law conflict was neutralized to some extent by family solidarity. This result further suggests that the tipping point of the female trade-off between staying with the natal family and leaving the natal family to join an economically well-established in-law family might have been reached very quickly among women living under the socioeconomic conditions of the Krummhörn region.

## Introduction

Humans typically organize not just their productive activities, but their reproductive activities within the framework of cooperative kin networks. Many scholars have argued that the motivational and emotional capacity to work together with family members is a key evolutionary adaptation that distinguishes humans from the other Great Apes [1]. The main focal points of the research to date on the reproductive consequences of the strategy referred to as “cooperative breeding” are bundled in with the question of to what degree members of kin networks help to increase fertility and child survival in their family by providing various kinds of support [2]. Indeed, an extensive literature has recently emerged that has examined the contributions of so-called “alloparents” to the successful reproduction of human breeders in a kin group [3]. These studies have suggested that alloparents play a crucial role in reproduction—and, ultimately, in the evolutionary success of humans [4].

Although the members of a breeding group rely on mutual assistance and support, aid and support are not granted unconditionally, but rather in accordance with each individual’s personal cost-benefit analysis. After all, by pursuing their own reproductive interests, alloparents are engaging in a more or less visible form of reproductive competition within their breeding group. Furthermore, alloparents face allocation problems. As their willingness to help is not unlimited, they are forced to make decisions regarding which family members they support, and under what terms. With respect to the scope and the quality of intrafamilial transactions, we can assume that spatial proximity plays an equally important role in the allocation of familial resources [5] and in varying levels of indigence [6].

Until now, the question of what effects cooperative breeding have on adult lifespans has hardly been studied. The possibility that the survival of an adult is also influenced by the size and the composition of his or her family network cannot be rejected *a priori*. The question that arises is whether the survival of reproducing females is affected under the operation of a cooperative breeding regime. With this analysis, we explore this possibility by examining mortality trends among reproductive women dependent on the composition of their kin network. Alleged effects of the family network hereby might be both behavioral-related as well as of structural nature. Empirically, it is an open question whether family members are more affected by social interaction with their kin or by the accompanying factors, such as socio economic status and resource access. In the following, we report what family and social factors are associated with mortality of reproductive women in the literature.

Women (and all female mammals in general) have higher reproductive mortality risks than men [7]. Although birth-related mortality has decreased dramatically in Western Europe over the past two centuries, maternal mortality in East Frisia was still rather high in the 18^th^ and 19^th^ centuries, at 152 deaths per 10,000 births [8]. Although 794 out of 10,000 East Frisian mothers died postpartum (Imhof [8] for the parish of Hesel, East Frisia), maternal mortality levels in this region were among the lowest in Germany. At that time, almost all women gave birth at home under the supervision of an experienced midwife. In the early post-natal period, in which both the mother and the newborn are dependent on outside help as a consequence of birth- and pregnancy-related strains, kin support can be expected to play an important role in mothers’ well-being and survival. It is known from medical and from epidemiological studies that experienced older women in particular tend to help enormously in preparations for birth and for delivery [9].

Studies have also shown that the family’s socioeconomic situation can have consequences for maternal mortality, i.e. female mortality during the first six weeks postpartum. In their review on global maternal mortality differentials, Ronsmans and Graham [10] pointed out that both large and small socioeconomic factors can affect maternal mortality. Similarly, in a study on several geographically separated villages in 18^th^- and 19^th^-century Germany, Scalone [11] found that local crop price fluctuations affected maternal mortality levels differently depending on the family’s social stratum.

However, there is very little empirical evidence in existing social-historical research indicating that historic maternal mortality trends were subject to family effects. For example, in their analysis of a historic population of Slavonia (Croatia, 1750–1898), Hammel and Gullickson [12] reported that the risk of maternal mortality was generally lower if a woman was living in a large patrilineal kin group, but that if the wives of her husband’s brothers were also present (the authors of the study call these wives “classic rivals”), her risk of mortality was elevated. Moreover, maternal mortality increased when the woman was married to one of the younger brothers. Thus, the existing behavioral ecology literature is still rather uninformative on this issue. The general methodological problem of kin studies is that behavioral-related kin effects might be disguised, moderated, or even compensated by structural effects. For instance, the presence of wife’s natal kin in the household is a potential scenario for behavioral-related kin effect, but might be more common for landless families in patrilocal populations which might suffer from limited resource access. Therefore in assessing the potential behavioral-related effects of kin it is essential to include structural characteristics of family, such as level of spatial of proximity between kin, and wealth. Since these structural effects might be highly context-specific, forecasts and predictions what kinds of kin effects are to be expected are very difficult.Moreover, husband’s kin might not only be less supportive to the mother,but also invest less in her children. Such lineage differentials in kin investment have long been recognized in the literature and are often interpreted as an expression of paternity uncertainty [14, 15, 16, 17, 18, 19, 20, 21]. However, the child investment by husband’s family may differ over the social contexts. If the wife lives in a house in which she is surrounded by her in-laws, paternal certainty may be assumed and therefore investment into her children might be higher than in social environment with less control.

However, paternity uncertainty is not the only factor that generates a lineage asymmetry in intrafamilial cooperation, as it is obvious that economic factors also affect intrafamilial transactions. The questions of which lineage generates resources and of how the distribution of these resources is regulated are particularly salient. These economic considerations can loom so large that they render the paternity uncertainty effect irrelevant at the behavioral level. These patterns can result in a “patrilateral bias,” such as the one found by Pashos [22] for rural Greece. Within a strong patriline, the investment in a son’s children seems to be more beneficial than the investment in the children of a daughter who lives in another, and possibly far away family. A woman in a patrilineal system pays for access to the resources belonging to the kin group of her husband by sacrificing proximity to her natal family. Under polygynous conditions, the costs can increase: i.e., the woman may face strong female-female conflicts [23, 24], and thus an increased mortality risk. For these reasons, it is important that we differentiate in our analysis between all kin (both natal kin and in-laws) and family members who were alive and were present in the daily life of the individual, and thus lived in the same household or parish. For direct behavioral interactions to have occurred, a minimum level of spatial proximity was needed.

Female-female conflicts are not restricted to reproductive individuals, but can also develop across generations [22]. In particular, a mother-in-law may have conflicts with her daughters-in-law. Voland and Beise [25] argued that under certain socioeconomic conditions, a mother-in-law’s interest in the productive work performed by her daughter-in-law may lead the older woman to exploit the younger woman, and that the mother-in-law’s motivation to care for her daughter-in-law is subordinate to this primary interest. There is also some evidence that the husband (and his kin, especially his mother) has an increased interest in the fertility of his wife [26, 27, 28, 29, 30], which, in addition to the risk of economic exploitation, generates a risk of reproductive exploitation. Both forms of exploitation could lead to increased maternal mortality, because the costs of maternal mortality are higher for the natal lineage than for the in-law lineage due to the differing degrees of genetic relationship. A deceased daughter cannot be replaced, whereas it may be possible to replace a deceased daughter-in-law. It remains to be investigated whether this structural “in-law conflict” [31] also has functional equivalents in the relationships of other genetically unrelated individuals within the family.

Nevertheless, even related females in extended matrilineal families are affected by the problem of limited intrafamilial resources. Therefore, reproductive competition among the women of a matrilineal kin group cannot be reduced to zero, despite the high average coefficient of relatedness of the relationship [32]. After all, the female family members are all dependent on the resources generated by the family, and the intragenerational transfer of resources is made by the mother to all of her daughters. Accordingly, Ji et al. [33] found in their analysis of the matrilineal Musuo (China) that the more female family members there were in a household, the more reproductive success (measured in fertility parameters) was reduced; and that competition between female cousins could be more extreme than competition between sisters. Sear’s [34] study of a population in Malawi also showed that competition between related women had clear effects, as the presence of maternal kin was associated with increased infant mortality.

The reconstitution of the families in the Krummhörn region (East Frisia, Germany) during the 18^th^ and 19^th^ centuries serves as the data basis for our study. Previous analyses of this material recognized the cooperation/competition conditionality within families [25, 27, 35, 36, 37, 38], but did not focus on the life chances of reproductive women. The Krummhörn population had a patrilineal (and, frequently, a patrilocal) structure, which was reflected in the cultural practice of patronymic name choices [39]. With respect to parameters of maternal mortality, we ask whether a patriline had a role in kin support, which may have overridden the evolutionary “default setting” of a kin-selected matrilineal bias in the well-being and survival of mothers. Since unlike landless workers and craftsmen, relatively prosperous large-scale farmers were able to transfer resources, this social group may be of particular empirical importance for our analysis. We therefore investigate the question of whether wealth (here in the form of land ownership) plays a moderating role in the models. This approach is theoretically motivated by the local resource competition model [40, 41], which has been proven to have substantial explanatory power in previous studies of the Krummhörn population. We therefore investigate further whether the local resource competition model is able to explain mortality differentials of reproductive females in addition to parameters of infant and child mortality [42] and the probability of dispersal [36].

In sum, we expect to find a conglomerate of kin effects which consist of behavioral-related as well as of structural effects. One central innovation of this study is therefore to disentangle behavior-related and structural kin effects by taking spatial proximity of kin into account (see Data and methods section on Modelling kin effects). Because of structural kin effects and the context-specific nature of behavior-related kin effects, this study employs an explorative approach, assessing influences of individual members of the natal as well as of in-law family and additionally the effect of the sizes of the lineages on the mortality of reproductive females.

## Data and methods

### Study population and period

Our data are derived from a family reconstitution study based on Protestant church registers and tax rolls of the Krummhörn region in East Frisia (Germany) from the 18th and 19th centuries. The historical Krummhörn was divided into 33 neighboring parishes, all of which are included in the dataset. The dataset contains 118,778 individuals who were in 34,708 marriages. It is archived at the GESIS-Institute (Cologne) with the label *ZA8630* (http://dx.doi.org/10.4232/1.12643). A comprehensive description of the database can be found here: http://www.eckart-voland.de/Research.html

Many of the records dated before 1720 are incomplete, and families from the social and economic elite tend to be overrepresented in these early records. After 1874, the church was no longer responsible for maintaining records of births, deaths, and marriages, as this task had been transferred to the civil registry offices (“Standesämter”). Because of the bias in the early records and the censoring after 1874, we decided to limit our analysis to females who were born to couples who had been married between 1720 and 1850. See Table 1 for the sample selection criteria and the descriptive statistics of the final sample.

**Table 1:** Descriptive statistics: Number of cases and failures, and mean ages at important events.

N girls born to marriages contracted between 1720 and 1850	**20,291**
N cases deleted because of missing info. on parents’ start and end dates of marriage	-6,473
N cases deleted because ID never married or the date of marriage is unknown	-8,130
N cases deleted because ID’s age at first marriage was higher than 45	-49
N cases deleted because ID married after January 1, 1874	-115
N cases deleted because ID out-migrated immediately after marriage	-610
N cases remaining in the sample	**4,914**
Born to N families	3,201
N died before reaching age 45	922
N died within a postpartum period	182
1st birth related	62
2nd birth related	28
3rd birth related	20
4th and higher birth orders	72
Mean age at death of IDs who died before reaching age 45 (standard deviation)	34.59 (±6.39)
N censored before reaching age 45	1,578
Mean age at censoring of these IDs (standard deviation)	35.17 (±6.20)
N IDs who survived to age 45	2,414
Total N episodes (on average per ID)	178,636 (36,35)
Mean age at first marriage = mean age at entry] (standard deviation)	25.99 (±4.59)
Mean age at exit (standard deviation)	39.10 (±6.50)
N IDs who experienced the death of the 1st husband before the age of 45	660
N IDs who married a 2nd time before the age of 45	318
N IDs who experienced the death of the 2nd husband before the age of 45	32
N IDs who married a 3rd time before the age of 45	15
N IDs who experienced the death of the 3rd husband before the age of 45	1
N IDs who married a 4th time before the age of 45	1
Birth cohort*	
1720–9	9
1730–9	123
1740–9	276
1750–9	296
1760–9	282
1770–9	397
1780–9	392
1790–9	506
1800–9	582
1810–9	530
1820–9	683
1830–9	473
1840–9	287
1850–9	78
Birth order (1 = first born)	
1	1,158
2	966
3	804
4	655
5	507
6	365
7	219
8	128
9	56
10	26
11	12
12	9
13	6
14	2
15	1

*—as used in the models

Geographically, the region was bordered to the north and west by the North Sea; to the south by the River Ems; and to the east by sandy soil and moorlands, which were impenetrable at that time. The Krummhörn region itself had very fertile marsh soil that was suitable for raising both crops and livestock. The settlement of the area had been completed in the late medieval period [39], and there was no significant population growth during the study period [43]. As the region was a saturated habitat with a finite amount of arable land, the population faced local resource competition [42]. Because access to land was limited, a stratified social structure arose among the population of the Krummhörn. The large-scale farmers with capital and status were at the top of this social hierarchy, while the small-scale farmers, craftsmen, and landless workers occupied the lower end of this social structure. About 70 percent of the families in the 18th century had either no land at all or farms too small to ensure subsistence, and were thus forced to supplement their income by working for the large-scale farmers [44]. Although there are no records indicating that the region was affected by famine or war during this period, as in all other parts of Europe, smallpox and other infectious diseases took a significant toll on the people of the region over the course of the 18th century [45]. The average family size was about four children [42, 44]. The families of the region practiced a form of ultimogeniture in which the youngest son inherited the undivided farm from the father [38]. All of the other offspring had to be compensated, often with cash. A daughter could expect to receive half as much as a son. As a consequence of these social institutions, families in the Krummhörn region tended to be relatively small, and the average age at first marriage was high [44]. Thus, late reproduction and low birth rates were the norm.

### Modelling kin effects

We use Cox regression [46, 47] to model the life course of reproductive females from the date of their first marriage to the age of 45. Therefore, all women were married at the start of observation, but dependent on their husbands’ survival they could have experienced episodes of widowhood and/or of remarriage within the study age range (see below). We choose date of first marriage as start of observation, because reproduction took place almost entirely within marriages. The age of 45 is widely used in female life course studies as an average age of menopause. Being reproductive in this context means that these women were at least once married before the age of 45. In estimating the kin effects on the mortality of reproductive women, we rely on a combination of models adjusted by clustering at the family level, and models stratified at the family level (family fixed effects) [48]. The former models investigate the general association between having kin and mortality among reproductive females, and thereby estimate the net result of kin effects. The latter models estimate likelihood functions with separate terms for each of the families in the dataset, and thus allow each family to have their own individual baseline hazard function. The key difference between the stratified and the clustered Cox regression models is that the stratified models identify kinship effects using the variation within families, but not between families. These stratified models control for unobserved heterogeneity if these factors were shared by sisters. By comparing the results of the clustered with the results of the stratified models we try to disentangle kin effects which were attributable to common causes from those which were directly linked to family members’ behavior or accompanying factors. For example, having a large number of siblings could have been associated with reduced mortality due to parental characteristics (common cause) such as parental skills and the quality of the household, and not because of direct interactions between siblings. A similar approach has been successfully applied in the comparison of the effect of having siblings in this study population and in the St. Lawrence valley (New France) [37]. However, one disadvantage of the fixed-effect approach is that the models exclude singlets (in our case IDs without any reproductive sister in the dataset) from the analysis. Dependent on the structure of the data, the number of cases is therefore often substantially smaller in the fixed-effect version when compared to the clustered model version. Thus, if there are inconsistent findings in both model versions, it has to be tested whether this is due to the exclusion of cases or due to the different estimation of the likelihood function. This could be shown by re-running the cluster model versions with exact the same number of cases which are included in the fixed-effect approach.

The level of genetic relatedness might matter for family relationships. We therefore include information about the presence (see below) of each individual’s natal core family, which consisted of mother, father, sisters, brothers, and offspring and natal extended family, which consisted of maternal and paternal aunts and uncles and their offspring (first cousins). We also include information about the presence of in-law relatives, who can be further identified as core family members (mother-, father-, sisters-, and brothers-in-law) or as extended family members. The time-varying data on the different individual family members are coded as dummy covariates. Each change in the kin composition (birth or death of an individual family member) is an event which brings a new episode of observation to the model. These linkages result in a large data setup; on average, there are 36 events for each woman between the date of her first marriage and the date of her exit from the sample (upon surviving to age 45 or prior death). Effects of kin belonging to the core or extended natal family are estimated based on all episodes, even if a woman was widowed or remarried. The impact of the in-law kin is, however, estimated only during a woman’s first marriage. Episodes after the husband’s death are excluded from the analysis, as it is unclear how the relationship between the reproductive woman and her in-law kin would have been affected by her husband’s death or by her remarriage.

Since we are interested in analyzing both behavior-related kin effects that arise from direct social interaction and non-behavior-related (structural) kin effects, we need to disentangle these two types of kin effects. We would like to know whether the supposedly positive effect of being a member of a large family was the result of having a supportive and functional kin network, or was merely a reflection of the family’s socioeconomic status. As behavioral effects applied only to family members with a certain level of spatial proximity, while structural kin effects did not require spatial proximity, we have created two sets of models to determine the significance of spatial proximity for kin effects. In the first set of models, we consider all living relatives, regardless of where they were residing. In the following, these models are referred to as “*alive models*.” In a second set of models we include only relatives who were living in the same parish as the individual of interest. In the following, these models are referred to as “*spatial models*.” In the spatial models, we assume that family members engaged in daily social interactions that had different effects on female mortality. In other words, for each woman and at every age, the alive models are able to determine how many relatives were alive, whereas the spatial models are able to determine whether these kin were living in the same parish. For all of the different models estimated, we include a set of covariates that control for potential confounding conditions based on the context into which a woman was born and was living. The primary variables of interest are those for kinship formation. The rest of the covariates are included because they may be correlated with both the dependent outcome and kin formation. These potential confounders are discussed in the paragraph below.

Women are especially vulnerable during postpartum periods (42 days after the birth). We therefore include a time-varying dummy covariate which indicates exposure to postpartum periods. We also include individual’s birth cohort, which is coded in decades, to control for changes in the population over time, and for the individual’s birth rank [49]. The married women are categorized into five groups based on their husbands’ land ownership status. Families who owned more than 75 *grasen* (10 grasen = 3.6 hectare) are classified as “large-scale farmers”, families who owned between 10 and 75 *grasen* are assigned to the “mid-scale farmers” group, while families who owned less than 10 *grasen* are categorized as “small-scale farmers.” Families who had no land property are classified as “landless,” and families for whom the level of land ownership was unknown are placed in the “unknown” group. The borders between these categories are more or less arbitrary, but fit well into the historical context [44, 50]. Finally, we include time-varying information on the number of living sons and daughters and on the total number of births, which might have been higher than the number of living children due to offspring mortality.

Including a large number of variables in statistical models increases the risk of model overfitting and of collinearity. In regard to kin effects, collinearity might be an issue when the existence of one kin is dependent on the existence of another (endogeneity), making it difficult to identify the effects separately. In our study collinearity problems exist if information on first cousins and their parents, who are aunts or uncles to the individual of interest, are included in the same model. For this reason, we estimate the effect of aunts, uncles, and their children (first cousins) in separate models. However, the problem of collinearity might still pose a problem for covariates which are not as directly connected. One strategy to avoid these risks might be to estimate the effect of the individual kin in separate models. This approach, however, could result in a bias due to omitted covariates. To approach this problem, we first run models that include all kin of the core family and alternate information on first cousins, aunts, and uncle (see “full models” S1, S3, S5, and S7 Tables). Then, in a second step, we run slimmed down versions of the models where the effect of the individual kin is estimated with the above mentioned potential confounders in a separate model (see “half models” S1, S3, S5, and S7 Tables). In a third step, we estimate the effect of the individual kin a separate model without any other kin information (see “simple models” S1, S3, S5, and S7 Tables).

All analyses had been performed in STATA 14.

## Results

We address our research questions using three different levels of analysis. In a first step, we investigate the association between natal and in-law kin and the mortality of reproductive women by applying clustered and fixed-effect models. The members of the natal family could be subdivided into a core family (1) consisting of mother, father, sisters, and brothers; and into an extended family (2) consisting of paternal and maternal aunts and uncles and their children (first cousins). The members of the in-law family were further identified as belonging to the husband’s core family (3) consisting of mother-, father-, sisters-, and brothers-in-law; or to the husband’s extended family (4) consisting of the mother- and the father-in-law’s brothers and sisters and their children. Due to the lack of space, the results of the covariates of interest are presented in Tables 2 and 3 without the results of the control covariates. The significant findings of the models are summarized in Table 4. The results of the full model are given in S1, S3, S5, and S7 Tables. Detailed descriptive statistics for these models are given in S1, S3, S5, and S7 Tables. In a second step, we estimate the absolute sizes of the lineages; and in a third step, we estimate the relative sizes of the lineages as they affected maternal mortality.

**Table 2:** Results of the Cox regression estimating the impact of blood kin on the mortality of reproductive women. Hazard ratios are presented together with indicators of statistical significance (** p<0.01, * p<0.05, + p<0.1). Robust standard errors are given in parentheses. All models control for ID’s birth cohort, birth order, marital status (husband alive), postpartum period, number of births, number of living offspring, and socio-economic status of the current marriage. Full models are presented in S1 and S3 Tables.

	Spatial^1^		Alive^2^	
	Clustered^3^	Fixed-effect^4^	Clustered^3^	Fixed-effect^4^
**Mother**	0.801*	0.692	0.874+	0.919
	(0.078)	(0.185)	(0.062)	(0.292)
**Father**	1.130	0.916	0.979	0.969
	(0.116)	(0.275)	(0.075)	(0.308)
**Sisters**	0.951	1.564**	0.968	4.912**
	(0.061)	(0.256)	(0.039)	(1.018)
**Brothers**	1.003	0.831	0.932+	0.345*
	(0.056)	(0.150)	(0.037)	(0.152)
**Maternal aunts**	1.104	0.988	1.090	0.914
	(0.134)	(0.414)	(0.066)	(0.339)
**Sons of maternal aunts**^5^	1.083	0.837	1.032	1.334
	(0.085)	(0.181)	(0.035)	(0.500)
**Daughters of maternal aunts**^5^	1.032	0.999	1.009	0.359*
	(0.088)	(0.341)	(0.037)	(0.169)
**Maternal uncles**	1.015	1.197	0.908	0.367*
	(0.113)	(0.440)	(0.065)	(0.157)
**Sons of maternal uncles**^5^	0.973	1.158	0.978	0.594
	(0.072)	(0.262)	(0.037)	(0.249)
**Daughters of maternal uncles**^5^	1.053	1.662	0.908*	0.504
	(0.080)	(0.596)	(0.039)	(0.313)
**Paternal aunts**	1.166	1.056	1.085	1.145
	(0.167)	(0.403)	(0.073)	(0.698)
**Sons of paternal aunts**^5^	1.139	1.136	0.976	0.920
	(0.097)	(0.290)	(0.040)	(0.551)
**Daughters of paternal aunts**^5^	1.007	1.058	0.978	1.247
	(0.118)	(0.320)	(0.043)	(0.662)
**Paternal uncles**	1.061	1.569	0.946	1.111
	(0.140)	(0.701)	(0.072)	(0.609)
**Sons of paternal uncles**^5^	0.939	0.953	0.923*	0.727
	(0.074)	(0.207)	(0.036)	(0.445)
**Daughters of paternal uncles**^5^	0.988	0.705	1.026	0.288
	(0.083)	(0.197)	(0.039)	(0.239)
**N women**	4,914	2,908	4,914	2,908
**Dead**	922	535	922	535
**N families (cluster and strata, respectively)**	3,201	1,195	3,201	1,195
**Observations**	178,636	114,353	178,636	114,353
**Log pseudolikelihood**	-7107.63	-284.19	-7104.85	-246.76

^1^ –dummies only consider living kin who were residing in the same parish as the ID

^2^ –dummies consider all living kin regardless their place of residence

^3^ –each individual is compared to all other reproductive women in the sample

^4^ –each individual is compared to her reproductive sisters

^5^ –Due to the problem of collinearity, the hazard ratio has been estimated in a separate model

**Table 3:** Results of the Cox regression estimating the impact of in-law kin on the mortality of reproductive women. Hazard ratios are presented together with indicators of statistical significance (** p<0.01, * p<0.05, + p<0.1). Robust standard errors are given in parentheses. All models control for ID of interest’s birth cohort, birth order, marital status (husband alive), postpartum period, number of births, number of living offspring, and socio-economic status of the current marriage. Full models are presented in S5 and S7 Tables.

	Spatial^1^		Alive^2^	
	Clustered^3^	Fixed-effect^4^	Clustered^3^	Fixed-effect^4^
**mother-in-law**	0.643**	0.909	0.866	0.928
	(0.079)	(0.241)	(0.078)	(0.189)
**father-in-law**	1.087	0.849	1.132	1.014
	(0.133)	(0.243)	(0.108)	(0.231)
**sisters-in-law**	0.970	1.234	0.967	1.012
	(0.067)	(0.199)	(0.045)	(0.110)
**brothers-in-law**	0.932	0.862	0.933	0.894
	(0.061)	(0.130)	(0.044)	(0.097)
**sisters of mother-in-law**	0.996	0.961	0.986	1.289
	(0.167)	(0.444)	(0.072)	(0.235)
**male children of sisters of mother-in-law**^**5**^	1.000	0.608+	1.015	0.899
	(0.097)	(0.166)	(0.041)	(0.100)
**female children of sisters of mother-in-law**^**5**^	1.141	1.052	1.109**	1.062
	(0.097)	(0.234)	(0.036)	(0.113)
**brothers of mother-in-law**	1.086	0.524+	1.003	0.578*
	(0.166)	(0.196)	(0.088)	(0.137)
**male children of brothers of mother-in-law**^**5**^	0.997	0.740	1.002	0.991
	(0.094)	(0.232)	(0.044)	(0.116)
**female children of brothers of mother-in-law**^**5**^	0.916	0.724	0.947	0.947
	(0.120)	(0.210)	(0.053)	(0.121)
**sisters of father-in-law**	1.477*	1.271	1.023	1.070
	(0.232)	(0.387)	(0.095)	(0.197)
**male children of sisters of father-in-law**^**5**^	1.187*	1.070	1.011	1.032
	(0.095)	(0.197)	(0.060)	(0.110)
**female children of sisters of father-in-law**^**5**^	1.025	0.907	1.032	1.041
	(0.115)	(0.226)	(0.063)	(0.129)
**brothers of father-in-law**	0.767	0.950	0.857	0.848
	(0.139)	(0.444)	(0.091)	(0.197)
**male children of brothers of father-in-law**^**5**^	1.016	1.134	0.982	0.976
	(0.095)	(0.244)	(0.046)	(0.104)
**female children of brothers of father-in-law**^**5**^	0.944	1.552	0.994	1.129
	(0.104)	(0.449)	(0.048)	(0.111)
**N women**	4,638	2,653	4,638	2,653
**Dead**	754	430	754	430
**N families (cluster and strata, respectively)**	3,085	1,100	3,085	1,100
**Observations**	162,755	106,980	162,755	106,980
**Log pseudolikelihood**	-6332.78	-244.39	-6341.01	-244.45

^1^ –dummies only consider living kin who were residing in the same parish as the ID

^2^ –dummies consider all living kin regardless their place of residence

^3^ –each individual is compared to all other reproductive women in the sample

^4^ –each individual is compared to her reproductive sisters

^5^ –Due to the problem of collinearity, the hazard ratio has been estimated in a separate model

**Table 4:** Summary of individual kin effects on the mortality of reproductive women. Effects of kin belonging to the extended natal and in-law families are only given (and printed in italics), if at least one model suggests that there is a significant (p<0.1) association.

Kin	Effect on the mortality of reproductive women		Interaction with SES^1^
	Kin is present in the parish	Kin is alive (not necessarily present in the parish)	
**Mother**	Reduces mortality	Tend to reduce mortality	No
**Father**	No significant effect	No significant effect	-
**Sisters**	Increase mortality Effect is only suggested by the family-fixed-effect model versions	Strongly increase mortality Effect is only suggested by the family-fixed-effect model versions	Yes (effect of present sisters among the large-scale farmers substantially weaker)
**Brothers**	No significant effect	Decrease mortality	No
***Daughters of maternal aunts***	*No significant effect*	*Decrease mortality* *Effect is only suggested by the family-fixed-effect model version*	-*
***Maternal uncles***	*No significant effect*	*Decrease mortality* *Effect is only suggested by the family-fixed-effect model version*	-*
***Daughters of maternal uncles***	*No significant effect*	*Decrease mortality when not present in the same parish* *Effect is only suggested by the clustered model version*	-*
***Sons of paternal uncles***	*No significant effect*	*Decrease mortality* *Effect is only suggested by the clustered model version*	-*
**Mother-in-law**	Reduces mortality Effect is only suggested by the clustered model version	No significant effect	Yes, effect is stronger among the large-scale farmers
**Father-in-law**	No significant effect	No significant effect	-
**Sisters-in-law**	No significant effect	No significant effect	No
**Brothers-in-law**	No significant effect	No significant effect	-
***Sons of sisters of the mother-in-law***	*Tend to decrease mortality* *Effect is only suggested by the family-fixed-effect model version*	*No significant effect*	-*
***Daughters of sisters of the mother-in-law***	*No significant effect*	*Increase mortality* *Effect is only suggested by the clustered model version*	-*
***Brothers of the mother-in-law***	*Tend to reduce mortality* *Effect is only suggested by the family-fixed-effect model version*	*Reduce mortality* *Effect is only suggested by the family-fixed-effect model version*	-*
***Sisters of the father-in-law***	*Increase mortality* *Effect is only suggested by the clustered model version*	*No significant effect*	-*
***Sons of sisters of the father-in-law***	*Increase mortality* *Effect is only suggested by the clustered model version*	*No significant effect*	-*

^1^—Socio-economic status

*—The results of the models investigating SES interaction of kin belonging to the extended families are bulky due to low sample size. An interpretation is therefore difficult. Please see also section 3.4

### Impact of the individual kin

#### Natal relatives

The clustered model suggests that there was an association between the presence of the mother and reduced mortality among reproductive women (hazard ratios: 0.801*). The corresponding fixed-effect model indicates an effect in the same direction (hazard ration 0.692) although the significance does not reach the 0.1 level (Table 2). The clustered model version which includes the same number of cases as the fixed-effect approach indicates that the exceeded level of significance in the fixed-effect approach is due the exclusion of singlets and not due to unobserved heterogeneity (hazard ratio 0.872; S1 Table). Interestingly, the effect of the mother is not significant in the alive models, which considered only whether the mother was alive, and not whether she was living in the same parish. This finding suggests that this was a behavior-related effect, and was not due to factors such as lifespan heritability. However, it appears that the presence of the father did not have a statistically significant effect, regardless whether he was living in the same parish.

The results of the clustered and the fixed-effect models are not in agreement regarding the effect of having sisters. Whereas the clustered models indicated that there was no association between having sisters and survival, the fixed-effect versions showed that having sisters was associated with a significant decrease in the likelihood of survival (hazard ratio in the fixed-effect spatial model: 1.564**; Table 2 & S1 Table). The alive model generated an even higher hazard ratio (4.912**; Table 2 & S3 Table), and further suggested that there was an effect in the opposite direction for having brothers (hazard ratio 0.345*; Table 2 & S3 Table). However, this brother effect was not statistically significant in the spatial models. We will revisit these findings when we discuss the wealth-related differentials of the kin effects on survival (see Result section on Wealth related kin effects).

The presence of uncles (brothers of the father), or aunts (sisters of the father or the mother) or of their children (first cousins) in the parish did not have statistically significant effects on the mortality of reproductive women. However, the alive models suggest that were some effects. The clustered model indicates that daughters of maternal uncles (hazard ratio 0.908*) and sons of paternal uncles were associated with reduced mortality (hazard ratio 0.923*). The corresponding fixed-effect model version shows that maternal uncles (hazard ratio 0.367*) as well as sons of maternal aunts decreased mortality (hazard ratio 0.359*)(Table 2 & S3 Table).

#### In-laws

The clustered spatial model found that the presence of the mother-in-law was associated with decreased mortality among reproductive women (hazard ratio 0.643**; Table 3 and S5 Table). The findings of the corresponding fixed-version model are generally in line with this result, although the hazard ratio was not statistically significant (hazard ratio 0.909). The effect was absent in the alive model, which suggests that it was attributable to either the mother-in-law’s behavior or another factor that was associated with her presence. We will revisit this finding when we discuss wealth-related differentials of the kin effects on survival (see Results section on Wealth related kin effects and Discussion). Opposite effects were observed for sisters of the father-in-law and their male children: their presence in the same parish was associated with decreased survival (hazard ratio 1.477* and 1.187*, respectively; Table 3 and S5 Table).

The results of the fixed-effect version of the spatial model further suggest that the presence of sons of the sisters and of the brothers of the mother-in-law tended to decrease mortality (hazard ratios 0.608+ and 0,524+, respectively; Table 3 and S5 Table). The corresponding fixed-effect alive model also found that the presence of brothers of the mother-in-law was associated with lower mortality (hazard ratio 0.578*). The results of the cluster alive model suggest that the presence of daughters of the sisters of the mother-in-law was linked to increased mortality (hazard ratio 1.109**; Table 3 and S7 Table).

### Absolute sizes of the lineages and mortality among reproductive women

In a first step, we estimated the sizes of the natal and of the in-law lineages simply by counting the family members (natal vs. in-law). We distinguished between kin who belonged to the core family and kin who belonged to the extended family. In a second step, we weighted these counts by the coefficient of genetic relatedness “r” (e.g., the count of sisters was multiplied by 0.5, whereas a first cousin was multiplied by 0.125) to determine whether the consideration of genetic relatedness is affecting the model outcomes. The results of models are given in Table 5 and S9 Table. Since we are comparing the sizes of lineages, we have to rely on the same episodes as for the estimation of the effects of the individual in-law kin (see Data and methods section on Modelling kin effects). The same applies to the estimation of the relative size of the lineages below.

**Table 5:** Results of the Cox regression estimating the impact of the absolute size of the lineages on the mortality of reproductive women. Hazard ratios are presented together with indicators of statistical significance (** p<0.01, * p<0.05). Robust standard errors are given in parentheses. All models control for ID of interest’s birth cohort, birth order, marital status (husband alive), postpartum period, number of births, number of living offspring, and socio-economic status of the current marriage. Full models are presented in S9 Table.

	Spatial^1^				Alive^2^			
	Unweighted (simple count)		Weighted (numbers are weighted with coefficient of relatedness)		Unweighted (simple count)		Weighted (numbers are weighted with coefficient of relatedness)	
	**Cluster-ed**^**3**^	**Fixed-effect**^**4**^	**Cluster-ed**^**3**^	**Fixed-effect**^**4**^	**Cluster-ed**^**3**^	**Fixed-effect**^**4**^	**Cluster-ed**^**3**^	**Fixed-effect**^**4**^
**Natal core family**	0.968	0.986	0.936	0.948	0.963	2.029**	0.929	4.147**
	(0.026)	(0.067)	(0.051)	(0.130)	(0.022)	(0.286)	(0.043)	(1.171)
**Natal extended family**	1.016	0.994	1.090	1.127	0.995	0.837	0.969	0.473
	(0.018)	(0.056)	(0.100)	(0.344)	(0.008)	(0.100)	(0.046)	(0.234)
**In-law core family**	0.895**	0.961	0.807**	0.940	0.951*	0.965	0.910*	0.953
	(0.029)	(0.073)	(0.052)	(0.144)	(0.023)	(0.058)	(0.043)	(0.114)
**In-law extended family**	1.020	0.956	1.076	0.715	1.005	1.005	1.005	0.951
	(0.021)	(0.046)	(0.126)	(0.194)	(0.010)	(0.022)	(0.058)	(0.128)
**N women**	4,914	2,908	4,914	2,908	4,914	2,908	4,914	2,908
**Dead**	833	494	833	494	833	494	833	494
**N families (cluster and strata, respectively)**	3,201	1,195	3,201	1,195	3,201	1,195	3,201	1,195
**Observations**	167,699	106,980	167,699	106,980	167,699	106,980	167,699	106,980
**Log pseudolikelihood**	-6339.73	-247.61	-6339.98	-247.24	-6342.29	-233.20	-6342.33	-233.07

^1^ –dummies only consider living kin who were residing in the same parish as the ID

^2^ –dummies consider all living kin regardless their place of residence

^3^ –each individual is compared to all other reproductive women in the sample

^4^ –each individual is compared to her reproductive sisters

The results of both, the unweighted and the weighted spatial models suggest that the presence of a large in-law core family was linked to decreased mortality among reproductive women (hazard ratios 0.895** and 0.807*, respectively). This pattern is also indicated by the clustered alive models (hazard ratio 0.951* and 0.910*, respectively). The results of the corresponding fixed-effect models did not agree with this finding. In these models we find that survival was not affected to a statistically significant degree by the absolute size of any type of family. The fixed-effect versions of both, the unweighted and the weighted alive models showed that having a large natal core family was significantly associated with increased mortality (hazard ratios 2.029** and 4.147**, respectively. The negative effect of the natal core family was attributable to the negative effect of having sisters (see Result section on Impact of the individual kin). A negative effect of the natal core family was not observed when sisters were excluded from the model (data not shown).

### Relative sizes of the lineages and mortality of reproductive women

The prevailing pattern of patrilocality within the Krummhörn region often resulted in a woman having no natal kin living close to her place of residence after she married. In such cases, reproductive women were especially likely to have been exposed to their in-law kin’s interests and strategies. The question is whether this situation affected women’s mortality risk. To address this question, we categorized the life course of reproductive women into episodes in which (1) the natal lineage was larger than the in-law-lineage, (2) the lineages were the same size, (3) the in-law lineage was larger than the natal lineage, (4) only natal kin were present, and (5) only in-law kin were present. We restricted the analyses to the spatial model, because some categories in the alive model were rare, and might therefore have generated wrong conclusions. For instance, category (4) or category (5) in the alive model would imply that there were no living natal or in-law family members, which would be of little relevance for the aim of this study.

The results of models that estimate the impact of the relative sizes of lineages are given in Table 6 and S10 Table. We have chosen category (4), *natal lineage only*, as the reference category. The models showed that there was no statistically significant association between having only in-law kin present in the parish of residence and increased mortality. The results were similar in cases in which the in-law family members outnumbered the natal family members. The findings of the weighted clustered model version suggest that mortality tended to be lower if the in-law lineage was larger than natal lineage (hazard ratio 0.781+) and that mortality was higher if the lineages were the same size (hazard ratio 1.503*).

**Table 6:** Results of the Cox regression estimating the impact of the relative size of the lineages on the mortality of reproductive women. Hazard ratios are presented together with indicators of statistical significance (* p<0.05, + p<0.1). Robust standard errors are given in parentheses. All models control for ID of interest’s birth cohort, birth order, marital status (husband alive), postpartum period, number of births, number of living offspring, and socio-economic status of the current marriage. Full models are presented in S10 Table.

	Spatial^1^			
	Unweighted (simple count)		Weighted (numbers are weighted with coefficient of relatedness)	
	Clustered^2^	Fixed-effect^3^	Clustered^2^	Fixed-effect^3^
**Relative sizes of lineages (Ref. natal lineage only)**				
**Natal lineage was larger**	1.078	1.274	1.118	1.263
	(0.128)	(0.370)	(0.128)	(0.354)
**Lineages were equal in size**	1.074	1.117	1.503*	2.201
	(0.181)	(0.418)	(0.297)	(1.101)
**In-law lineage was larger**	0.902	0.822	0.781+	0.703
	(0.122)	(0.258)	(0.103)	(0.206)
**In-law lineage only**	0.907	1.196	0.906	1.237
	(0.080)	(0.264)	(0.080)	(0.275)
**N women**	4,914	2,908	4,914	2,908
**Dead**	833	494	833	494
**N families (cluster and strata, respectively)**	3,201	1,195	3,201	1,195
**Observations**	167,699	106,980	167,699	106,980
**Log pseudolikelihood**	-6345.03	-247.62	-6341.04	-245.72

^1^ –dummies only consider living kin who were residing in the same parish as the ID

^2^ –each individual is compared to all other reproductive women in the sample

^3^ –each individual is compared to her reproductive sisters

The finding that reproductive women did not have higher mortality risks if there many or exclusively in-laws kin in the parish raises two main issues. The first is that there may be a hidden wealth effect. A potential genetic conflict between the reproducing woman and her in-law relatives might be attenuated by the wealth of the in-law family. We will address this issue in the next section. The second issue is related to the marital status of the reproductive women. It is important to keep in mind that the estimates in Table 6 are based on episodes that took place during the woman’s first marriage. The relationship between the reproductive woman and her in-laws might substantially change after the death of her husband.

### Wealth-related kin effects

In order to investigate whether wealth moderated the kin effects identified in the analyses in Result section on the impact of the individual kin, we included interaction terms between the socioeconomic status (SES) (categories: large-scale farmers, landless, others, and unknown) of the woman’s current marriage and kin (coded as a dummy variable). We also tested the interaction between wealth and all kin, regardless whether analyses on the impact of the individual kin indicated significant general impacts. In these analyses, we did not find evidence that some kin effects are masked by the SES. Therefore, we decided to refrain from presenting these several dozen interaction models and only to present results for those kin who had a general impact on survival. However, the results of these models are available on request. The interactions were investigated in two different ways. In model (A) we estimated the effects of having kin in each SES category separately. This model series provided us with an overview of whether the kin effects differed across the SES categories. However, such an approach lacks a statistical test for whether the estimated kin effects differed significantly across the SES groups. We therefore created another series (B) in which we estimated the effects of having kin while using landless women as the reference category. These models provided p-values for the interaction terms that told us whether a kin effect was significantly moderated by SES.

#### Natal relatives

The results of the fixed-effect spatial models that investigated the interaction between having biological sisters in the same parish and SES suggest that having sisters increased mortality especially among reproductive women who belonged to the landless group (A: hazard ratio for large-scale famers 1.030; hazard ratio for landless 1.933**; B: hazard ratio * interaction term 1.955** * 0.549; S9 Table). In other words, the aforementioned (see 3.1.) negative effect of having sisters was substantially weaker among the wealthy elite.

#### In-laws

The results of the clustered spatial models (A) suggest that mortality risks among reproductive women were affected by the presence of the mother-in-law. (hazard ratio for large-scale famers 0.213; hazard ratio for landless 0.656*; S10 Table). It appears that the effect was stronger among the group of large-scale farmers (hazard ratio 0.7759^+^ * 0.323; S10 Table).

## Discussion

Our aim in this study was to answer the question of whether kinship composition affected mortality among reproductive women using three analytical levels. In a first step, we investigated the association between the presence of both natal and in-law family members and mortality among reproductive women; in a second step, we estimated the effects of the absolute sizes of the spousal lineages; and in a third step, we estimated the effects of the relative sizes of the spousal lineages. The challenge was to differentiate between “true” kin effects arising from behavioral interactions or accompanying factors on the one hand, and correlations attributable to common causes on the other. We therefore compared the results of clustered models with those of fixed-effect models.

The kin effects on mortality among the reproductive women studied might have been caused by at least two different mechanisms. First, there might have been effects that arose from direct social interaction. For a reproductive woman to have experienced both supportive and competitive interactions with family members on a daily basis, a certain level of spatial proximity between the woman and her kin was needed. We regard the positive effect of the biological mother (Table 2A and 2B) as an example of such a direct interaction. The presence of the mother (but not of the father) reduced the mortality risk of the reproductive daughter. This could be because mothers typically offer their daughters support in daily life, as well as in crises.

Scelza [51] also emphasized that the presence of the mother is important for the personal well-being of the daughter, and showed that there is a close relationship between a mother and her reproductive daughters, especially during pregnancy and during the neonatal period. It appears that simply having the option of seeing her mother is of great value for the reproductive daughter, and reduces her need to have her mother permanently present [52].

Second, in addition to the effects that arose from direct social interactions in daily life, there may have been kin effects that were caused by broader kin interactions. Siblings, and especially those of the same gender, tend to deploy and compete for the same resources within the family [33, 37, 53, 54, 55]. Family resources are not unlimited, and this constraint explains why mortality increased with the number of living sisters. Parents in the Krummhörn (and, indeed, in Quebec) appear to have reduced their per-daughter investment depending on their number of living daughters [37]. This finding also holds to a lesser degree for sons. As a consequence, female infant and child mortality was increased by the number of living sisters [37]. The results of our models suggest that the effect of the parental quality/quantity trade-off prolonged the adult lives of females. Thus, a reproductive woman with a large number of sisters had a higher mortality risk than a woman with fewer female siblings. A similar finding was reported by Donrovich et al. [53] for a population in Antwerp. They showed that having a large number of siblings was associated with increased mortality after age 50. However, having sisters might have also had a positive behavior-related effect, which explains why having sisters who were living in the same parish was less harmful to all of the living sisters. In other words, the negative effect of having sisters was offset to some extent by the support provided by the sisters who were living in the same parish. It is possible that the sisters’ ages influenced whether these interactions were more competitive or more cooperative [56, 57]. Interestingly, we found an interaction effect between having sisters and socioeconomic status. Having a large number of sisters was associated with lower survival chances among the landless, but not among the large-scale famers. This finding supports the hypothesis that the reproductive quality/quantity trade-off within landless families was much more pronounced.

It might appear surprising that we found hardly any effects for kin who belonged to the woman’s extended natal family. In reality, these extended family members may have offered conditional support on many occasions. Whereas the positive effect of having the mother present during crises is apparent, transactions involving other kin might have run in the background. Situational family support was likely contingent upon the number of potential helpers available during a crisis period. It therefore appears that we should take the term “family networks” literally. Like knots in a fishing net, family members cannot be ranked according their importance. However, the women who were in the process of childbearing could not participate in this helper pool. Thus, their support was not conditional in the same way as the support provided by (potential) helpers. Such an interpretation is consistent with the results of our models.

The most interesting finding of this study is that the mother-in-law is not associated with increased mortality. In fact, the clustered model version suggests a positive effect, whereas the family fixed version indicates a more or less neutral effect of living in close distance with the mother-in-law. Based on the findings of previous studies on the effects mothers-in-law have on stillbirths and infant mortality [25, 35, 36, 38], our recent finding appears to be counterintuitive. This apparent contradiction is, however, resolved when we consider that the positive mother-in-law effect was mainly observed among large-scale farmers, whereas the findings regarding the impact of mothers-in-law on stillbirths and infant mortality are based exclusively on observations of women in the landless group. Thus, this effect appears to be social-stratum-specific. We believe that this effect can be explained by the prevalence of consanguineous marriages among the large-scale farmers in the Krummhörn. A study in preparation found that the level of consanguinity is higher among landowning families. In particular, consanguinity among families of the large-scale farmers was about three times higher when compared to the landless [58]. First cousin marriages, double marriages (e.g. two brothers of one family marry two sisters of another), and marriages between uncles and nieces reflected strategies of property concentration and the establishment of reliable support in social affairs with the aim of securing political hegemony [59], Consangunity was a preference within the group of large-scale farmers with a long tradition. In the long run, it resulted in a substantial increase in the inbreeding coefficient.

For instance, a mother-in-law may have also been a biological aunt following a first cousin marriage. This blood relationship may have compensated for or neutralized the costs of the genetic in-law conflict. On the proximate level, the tendency to exploit the daughter-in-law that was observed among the landless families [25] might have been attenuated or even entirely superseded in the wealthy families by the inclination to provide care and support. Within the social stratum of landless workers and rural tradesmen, consanguineous marriages were less frequent, and the resulting lack of genetic proximity between the mother-in-law and the daughter-in-law might explain the absence of a protective mother-in-law effect.

Interestingly, a similar effect, albeit less pronounced, was found among the daughters of the mother-in-law. As those sisters-in-law may have also been the woman’s first cousins, this pattern suggests that there could have been a continuation of nepotistic tendencies across the in-law rift. However, the presence of sisters of the mother-in-law had no apparent effect. While these women may have also been the biological aunts of the individual of interest, unlike the mother-in-law, they were not the grandmother of her children. It seems that this distinction was associated with a reduction in the motivation to provide intra-family help. These findings in turn demonstrate how sensitively cooperation and rivalry were balanced within kin networks, and how relevant the type of relationship was in the regulation of family transactions.

We can assume that the confounding of wealth and kin effects is a pattern that occurs not just among the historical population of the Krummhörn region. We would expect to find similar conditions in every population in which agrarian or aristocratic families pursue wealth and power concentration via marriage strategies. Consanguineous marriages might also result in an increased F-value among the natal families of fathers-in-law. However, the effects of this in-law group appear to be rather neutral. We could only speculate that the differences between the mother-in-law and her kin and the father-in-law and his kin reflect paternity uncertainty.

In our view, the results regarding the effects of the absolute and the relative sizes of the lineages on maternal mortality raises two interesting issues. First, a reproductive woman who had a large number of natal family members living in the same parish did not necessarily have a decreased mortality risk. Second, the natal environment was not necessarily the best environment for reproductive women.

It is striking that a reproductive woman who lived exclusively or predominately with members of her in-law lineage did not face a higher mortality risk than a woman who lived exclusively with her natal kin. We argue that the decision to marry a man from a large lineage was, on average, also a decision to marry into an economically well-established or socially powerful lineage. We should again emphasize that the positive in-law effect was mainly driven by large-scale farmers, and that a woman who moved away from her natal family to live with her husband’s family might have had higher socioeconomic expectations. Since these individuals succeeded in being accepted as a wife and a farmer by a wealthy family in a highly competitive environment, it may be expected that they had certain personal characteristics that might have been associated with greater vitality and less vulnerability. In short, a woman of higher quality was especially likely to have found a good environment for raising her children and to have joined her husband’s family, whereas a woman with fewer advantages was more likely to have stayed in close spatial proximity to her parents’ home.

Thus, the finding that mortality among reproductive women was not increased among stronger in-law lineages is at least a partial consequence of the social assortment of spouses. High-quality women succeeded in attaining high-quality positions. Unfortunately, the question of to what extent the reduced mortality among reproductive women was due to phenotypic quality, or to the childbearing environment, is hard to answer with the current study design. As a side note, it is worth mentioning that research on the origins of inequality faces the same problem. Biological and socioeconomic factors form a mélange of effects that are hard to disentangle [60].

Considering the relatively small advantages, especially for female landless laborers, associated with living in close spatial proximity to sisters, it may have made sense for women to have pursued a strategy of social selectivity or even of hypergamy. The tipping point in the trade-off between staying and leaving [23] could have quickly reached in the socioeconomic conditions of the Krummhörn region, because a woman’s sacrifice of proximity to her natal family could have relatively small compared to the benefits associated with having access to the resources of an economically well-established kin group in a more distant location. Although these considerations might be of speculative nature, they fit well with the “patrilineal bias” in grandmaternal investment described by Pashos [22] for an agrarian Greek population.

While certain questions remain open, we believe we can draw some general conclusions from our study. Our findings indicate that there were mortality differentials among reproductive women, and that those differentials were connected to the composition of the kin network. More specifically, our study found that the naïve assumption that a woman’s natal kin represented a source of support and that her in-law kin represented a source of competition falls short for four main reasons. First, there may have been resource competition among the offspring of a family that was conditional on the family’s resource situation. Second, both natal and in-law-kin had a common interest in the descendants of the marriage. This might be the reason why a woman could, despite the structural in-law conflict, develop a close emotional relationship with her in-law kin [61]. Third, a socioeconomically well-established in-law family might have provided a woman with more opportunities in life, and ultimately with more reproductive fitness, than a more cooperative, but weaker natal kin group. Fourth, the practice of concentrating property and social influence through consanguineous marriage might have neutralized in-law conflict strategies, thereby turning potential rivals into welcomed allies.

## Supporting information

S1 Table Spatial models estimating the effect of the individual natal kin. Summary of effects is presented in Table 2 in the Result section.
(XLSX)

S2 Table Number of cases for the models presented in S1 Table. (XLSX)

S3 Table Alive models estimating the effect of the individual natal kin. Summary of effects is presented in Table 2 in the Result section.
(XLSX)

S4 Table Number of cases for the models presented in S3 Table. (XLSX)

S5 Table Spatial models estimating the effect of the individual in-law kin. Summary of effects is presented in Table 3 in the Result section.
(XLSX)

S6 Table Number of cases for the models presented in S5 Table. (XLSX)

S7 Table Alive models estimating the effect of the individual in-law kin. Summary of effects is presented in Table 3 in the Result section.
(XLSX)

S8 Table Number of cases for the models presented in S7 Table. (XLSX)

S9 Table Spatial and alive models estimating the effect of the absolute size of the lineages. Summary of effects is presented in Table 5 in the result section.
(XLSX)

S10 Table Spatial models estimating the effect of the relative size of the lineages. Summary of effects is presented in Table 6 in the result section.
(XLSX)

S11 Table Spatial and alive models estimating the interaction between SES and the individual natal kin. (XLSX)

S12 Table Spatial and alive models estimating the interaction between SES and the individual in-law kin. (XLSX)
